# Internal Mammary Sentinel Lymph Nodes in Breast Cancer - Effects on Disease Prognosis and Therapeutic Protocols - A Case Report

**DOI:** 10.3889/oamjms.2015.025

**Published:** 2015-02-16

**Authors:** Sinisa Stojanoski, Nevena Ristevska, Daniela Pop-Gjorcheva, Borce Antevski, Gordana Petrushevska

**Affiliations:** 1 *Institute of Patophysiology and Nuclear medicine “Acad Isak S. Tadzer”, Faculty of Medicine, Ss Cyril and Methodius University of Skopje, Skopje, Republic of Macedonia*; 2 *University Clinic of Thoraco-Vascular Surgery, Faculty of Medicine, Ss Cyril and Methodius University of Skopje, Skopje, Republic of Macedonia*; 3 *Institute of Pathology, Faculty of Medicine, Ss Cyril and Methodius University of Skopje, Skopje, Republic of Macedonia*

**Keywords:** sentinel lymph nodes (SLN), internal mammary lymph nodes, gamma detection probe, metilen blue dye, breast cancer

## Abstract

**BACKGROUND:**

The main prognostic factor in early staged breast cancer is the axillary lymph node metastatic affection. Sentinel lymph node biopsy, as a staging modality, significantly decreases surgical morbidity. The status of internal mammary lymph nodes gains an increased predictive role in grading breast carcinomas and modulation of postoperative therapeutic protocols. If positive, almost always are associated with worse disease outcome. Nevertheless, the clinical significance of internal mammary lymph node micrometastases has not been up to date precisely defined.

**AIM:**

To present a case of female patient clinically diagnosed as T1, N0, M0 (clinical TNM) ductal breast carcinoma with scintigraphic detection of internal mammary and axillary sentinel lymph nodes.

**METHODS:**

Dual method of scintigraphic sentinel lymph node detection using 99mTc-SENTI-SCINT and blue dye injection, intraoperative gamma probe detection, radioguided surgery and intraoperative ex tempore biopsy were used.

**CASE REPORT:**

We present a case of clinically T1, N0, M0 ductal breast cancer with scintigraphic detection of internal mammary and axillary sentinel lymph nodes. Intraoperative ex tempore biopsy revealed micrometastases in the internal mammary node and no metastatic involvement of the axillary sentinel lymph node.

**CONCLUSION:**

Detection of internal mammary lymph node metastases improves N (nodal) grading of breast cancer by selecting a high risk subgroup of patients that require adjuvant hormone therapy, chemotherapy and/or radiotherapy.

## Introduction

Sentinel lymph nodes (SLN) are the first nodes of the lymphatic drainage pathway of malignant tumors, hence the first nodes in which cancerous cells could spread after they leave the primary tumor and enter the lymphatics. SLN detection and intraoperative ex tempore pathohistological evaluation, with high sensitivity and specificity, provide valid information about the tumor’s lymphatic spread (metastases / micrometastases) and enables mapping of the tumor’s lymphatic drainage. It also determines the type and extensity of the surgical intervention – conserving or radical mastectomy / lymphadenectomy. If SLN are free of micrometastases on ex tempore evaluation, sentinel lymph node biopsy (only the SLN removal) is performed. But, if metastases are detected, nodal up staging (from N0 to N1) is obligatory and radical lymphadenectomy is performed. Axillary lymph node status is the most important prognostic factor in breast cancer patients. The technique for SLN detection, extirpation and ex tempore evaluation is a minimally invasive operative procedure, but at the same time a highly precise evaluation method, for axillary lymph node metastatic involvement. The literature data indicates that this technique could be considered as a successful alternative to the conventional radical axillary lymph node dissection [1, 2].

Lately, the status of internal mammary lymph nodes increases its predictive role in breast carcinoma grading and modification of the postoperative therapeutic protocols [3]. If positive, they are almost always associated with worse disease outcome and therapeutic protocol redefinition [4]. Nevertheless, visualisation of the internal mammary lymph nodes with the method of SLN detection is rare, in only 20% of the cases, and the clinical significance of micrometastases in this lymphatic drainage pathway has not yet been precisely defined [5].

The aim of this study was to present a case of female patient clinically diagnosed as T1, N0, M0 (clinical TNM) ductal breast carcinoma with scintigraphic detection of internal mammary and axillary sentinel lymph nodes.

## Material and Methods

The scintigraphic SLN detection included preoperative subcutaneous application (16 h prior the surgical procedure) of 4mCi (150 MBq) 99mTc-SENTI-SCINT, subdivided in 4 separate doses (each dose of 1 mCi/37MBq respectively) injected into 4 separate periareolar locations. Considering the fact that radiotracers were used, we followed the ALARA principles (as low as reasonably achievable) in order to obtain the best diagnostic presentation with the minimum radiation burden to the patient.

SENTI-SCINT is a MEDI-RADIOPHARMA LTD Hungary commercial kit, composed of human serum albumin nano-sized colloid particles with diameter of 100-600 nm in a form of sterile lyophilised powder. The particles were labelled with 99mTc-pertechnetate.

Quality control of the radiolabeled tracer was performed with ascendant paper chromatography. The injected volume at single site did not exceed 0.2-0.3 ml, and it was extremely important to avoid the blood vessels during the application. In case of intravascular application, the SLN detection procedure is considered unsuccessful.

Post injection, we performed dynamic acquisition (30 minutes; 30 frames, 60 seconds per frame, 256 × 256 matrix) followed by static AP, AL (lateral) and AO (oblique) positions (300 seconds per position) 30 min, 1 h, 2 h and 16 h post injection, using the dual head gamma camera Mediso DHV Nucline Spirit. We used cobalt source Featherlite Co57 flood source MED 3709 for body contour drawing, and the SLN detection was performed with gamma detection probe EUROPROBE SYSTEM CE 0459. Preoperatively we used gamma probe to detect the SLN location, quantify and mark the skin site above it in 2 positions AP and AL/AO in order to help the surgeon to determine the best operation incision approach for minimally invasive surgical procedure. Intraoperatively, the gamma probe was used for radioguided surgery, activity quantification of the extirpated SLN and possible additional SLN or remnant detection.

The sensitivity and specificity of the SLN detection method was increased with the use of sterile solution of metilen blue dye (vital blue) 0.5-1 ml, injected intradermally 15 min before the operative procedure.

The histopathological evaluation of the extirpated SLN was performed by routine hematoxylin and eosin (H & E) staining and immunohistochemistry section analyses.

## Case Report

We present a case of a 48 years old female patient clinically diagnosed as T1, N0, M0 (clinical TNM) ductal breast carcinoma. The patient underwent diagnostic mammography (27.03.2013) and the results presented mastopathy with diffuse monomorphic calcifications. No pathological findings were detected (ACR type 4; BIRADS 2). Ultrasonography was performed the next day and it revealed a 1 cm hypoechoic formation, without pathological vascularization, in the left breast, in the lower medial quadrant, parasternal (BIRADS 4). There were no enlarged axillary lymph nodes on palpation or ultrasonography. Core biopsy was performed (29.03.2013) that showed presence of ductal breast carcinoma. The patient was admitted to our Institute (3.04.2013) for scintigraphic SLN detection. We used the dual method of scintigraphic SLN detection using 99mTc-SENTI-SCINT and blue dye injection.

**Figure 1:**
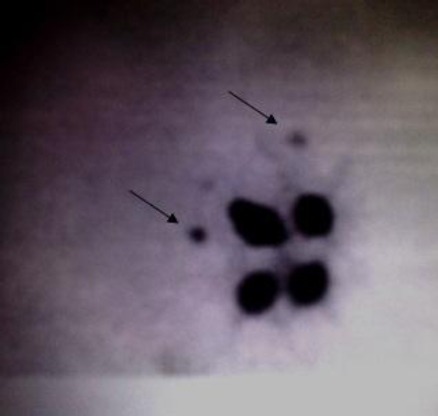
*Dual method of scintigraphic SLN detection using 99mTc-SENTI-SCINT and blue dye injection - AP position 2 h post injection*.

**Figure 2:**
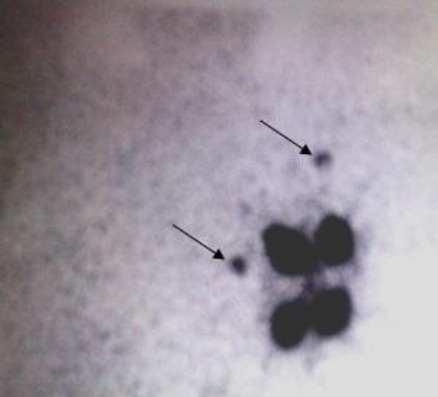
*Dual method of scintigraphic SLN detection using 99mTc-SENTI-SCINT and blue dye injection - AP position 16 h post injection*.

The results revealed double drainage of the radiotracer, primary towards the intramammary lymph nodes and secondary towards the axillary lymph nodes. We were able to detect 2 SLN, the first one in the region of a. mammaria interna and the second one in the axillary region. (Fig. 1 and Fig. 2) Drainage of the blue dye was towards the internal mammary region and no drainage towards the axillary region was observed.

Radioguided surgical procedure was performed and both SLN, the internal mammary and the one in the axillary region, were extirpated. Ex tempore pathohistological analysis presented no micrometastases in the axillary SLN and micrometastases > 0.2 mm in the internal mammary SLN. The final postoperative histopathological evaluation confirmed these results and the immunohistochemical findings presented ER+, PR-, HER2+. Taking this into consideration, and also the negative tumor markers, negative ultrasonography results and negative bone scan results, the postoperative TNM classification was pT1 N1b M0. The operative procedure included sentinel lymph node biopsy of both axillar lymph node and lymph node around arteria mamaria interna and quadrantectomy, without radical axillary lymphadenectomy, considering the fact that the SLN in the axillary region was negative on ex tempore pathohistological examination. Since the internal mammary SLN was positive for micrometastases, the patient was admitted to the University Clinic of Radiotherapy and Oncology for adjuvant treatment.

## Discussion

SLN detection technique was developed for precise diagnosis of micrometastases in the axillary lymph nodes in breast cancer patients with clinically and ultrasonographically negative lymph nodes staged as T1-2 N0 M0 [1, 6]. In the last years, the status of the internal mammary lymph nodes increases its predictive role in N (nodal) breast carcinoma grading and postoperative therapeutic protocols modification. The interest for this lymphatic pathway dates many years before the introduction of lymphoscintigraphy and SLN detection methods, when during the extended radical Halsted mastectomy the intramammary lymph nodes had also been extirpated and histopathologically analysed [7, 8]. This radical type of mastectomy enabled the first insight in the internal mammary lymphatic pathways and those first results also indicated that if positive for metastases, this lymph nodes correlate with worse disease outcome, more aggressive carcinoma variants and lower survival rate [9-11].

Internal mammary lymph nodes are visualised in 20% of the cases in which SLN detection method has been applied. Shen et al. in their study point out the fact that metastatically positive internal mammary lymph nodes are an independent factor for the decrease in survival rate [5]. Guth et al. and Hogan et al. in two independent research studies conclude that patients with positive internal mammary lymph nodes have more aggressive tumours, with higher rate of lymphovascular invasion and axillary metastases [4, 12].

Patients diagnosed with positive internal mammary lymph nodes and negative axillary lymph nodes, have similar 10 years survival rate, approximately 60%, as patients with positive axillary lymph nodes [6, 11]. This fact favours the necessity of internal mammary lymph node detection and one should always have in mind the possible drainage towards these lymphatic pathways in order not to avoid a high risk subgroup of patients with axillary negative and possible internal mammary positive lymph nodes.

Up to date clinical research trials consider the postoperative systemic adjuvant chemo and radiotherapy as an option in this subgroup of patients [13]. Effects of radiotherapy on survival rate in patients with positive internal mammary lymph nodes are subject of a large clinical trial (EORTC 22922). Considering the results of this study, patient selection for adjuvant radiotherapy should be based on a diagnosis of positive internal mammary lymph nodes with SLN detection method. In this manner, the diagnosis of positive internal mammary lymph nodes improves the N grading of breast carcinomas.

The dilemma if the positive internal mammary lymph nodes correlate with the status of the axillary lymph nodes is current clinical scientific issue. In our case both internal mammary and axillary SLNs were detected, the axillary being negative on ex tempore histopathology analysis, and the internal mammary being positive.

Previous scientific research in this field indicates different combinations of metastatic involvement in both lymphatic pathways: negative internal mammary and negative axillary, positive internal mammary and negative axillary and positive internal mammary and positive axillary lymph nodes (Table 1).

**Table 1:** Published papers about sentinel lymph nodes.

References	Patients with ISLN	Negative ISLN Negative ASLN	Positivei ISLN Negative ASLN	Positive ISLN Positive ASLN
Intra et al., 2009 [14]	15	9	6	
Kijima et al., 2008 [15]	4	2	2	
Mathelin et al., 2005 [16]	2	1	1	
Tytle et al., 2003 [17]	1			1
Gajdos et al., 2001 [18]	1			1

ISLN - intramammary sentinel lymph nodes; ASLN - axillary sentinel lymph nodes.

Up to date, no case of negative ISLN and positive ASLN has been reported in the literature. This fact raises the dilemma of possible primary breast carcinoma drainage towards the internal mammary lymph nodes and remains as a scientific challenge in future. In this context, Vijan et al. in their scientific research conclude that patients with positive ISLN have high incidence of axillary metastatic involvement and they raise a hypothesis about internal mammary lymph nodes being “sentinel nodes” for the axillary lymph nodes [19].

According to the scientific data, if internal mammary lymph nodes are positive and axillary nodes are negative, radical axillary dissection could be avoided. The extent of the surgical procedure concerning the axilla should be based only on the status of the axillary lymph nodes [20].

In conclusion, the clinical significance of the positive internal mammary sentinel lymph nodes has not yet been clearly and precisely defined. Further detailed analysis on larger scale case series will contribute to solving this clinical issue. Up to date, we can conclude that the diagnosis of the ISLN improves N breast carcinoma grading by detecting a subgroup of high risk patients that require adequate postoperative adjuvant chemo and radiotherapy. The clinical decision for the surgical treatment of the axilla should be based solely on the axillary lymph node status even if the internal mammary nodes are positive for metastases.
